# Incidence and Mortality of Acute Kidney Injury after Myocardial Infarction: A Comparison between KDIGO and RIFLE Criteria

**DOI:** 10.1371/journal.pone.0069998

**Published:** 2013-07-23

**Authors:** Fernando B. Rodrigues, Rosana G. Bruetto, Ulysses S. Torres, Ana P. Otaviano, Dirce M. T. Zanetta, Emmanuel A. Burdmann

**Affiliations:** 1 Division of Emergency and Chest Pain Center, Hospital de Base, Sao Jose do Rio Preto Medical School, Sao Jose do Rio Preto, São Paulo, Brazil; 2 Division of Nephrology, Hospital de Base, Sao Jose do Rio Preto Medical School, Sao Jose do Rio Preto, São Paulo, Brazil; 3 Division of Cardiology, Hospital de Base, Sao Jose do Rio Preto Medical School, Sao Jose do Rio Preto, São Paulo, Brazil; 4 Public Health School, University of Sao Paulo, Sao Paulo, Sao Paulo, Brazil; 5 Division of Nephrology, University of Sao Paulo Medical School, Sao Paulo, São Paulo, Brazil; Virginia Commonwealth University, United States of America

## Abstract

**Background:**

Acute kidney injury (AKI) increases the risk of death after acute myocardial infarction (AMI). Recently, a new AKI definition was proposed by the Kidney Disease Improving Global Outcomes (KDIGO) organization. The aim of the current study was to compare the incidence and the early and late mortality of AKI diagnosed by RIFLE and KDIGO criteria in the first 7 days of hospitalization due to an AMI.

**Methods and Results:**

In total, 1,050 AMI patients were prospectively studied. AKI defined by RIFLE and KDIGO occurred in 14.8% and 36.6% of patients, respectively. By applying multivariate Cox analysis, AKI was associated with an increased adjusted hazard ratio (AHR) for 30-day death of 3.51 (95% confidence interval [CI] 2.35–5.25, p<0.001) by RIFLE and 3.99 (CI 2.59–6.15, p<0.001) by KDIGO and with an AHR for 1-year mortality of 1.84 (CI 1.12–3.01, p = 0.016) by RIFLE and 2.43 (CI 1.62–3.62, p<0.001) by KDIGO. The subgroup of patients diagnosed as non-AKI by RIFLE but as AKI by KDIGO criteria had also an increased AHR for death of 2.55 (1.52–4.28) at 30 days and 2.28 (CI 1.46–3.54) at 1 year (p<0.001).

**Conclusions:**

KDIGO criteria detected substantially more AKI patients than RIFLE among AMI patients. Patients diagnosed as AKI by KDIGO but not RIFLE criteria had a significantly higher early and late mortality. In this study KDIGO criteria were more suitable for AKI diagnosis in AMI patients than RIFLE criteria.

## Introduction

Development of AKI has been consistently associated with a higher mortality rate in acute myocardial infarction (AMI) patients [1]–[10]. However, the lack of a uniform standardization for the diagnosis of AKI led to conflicting epidemiological data, troubling the advancement in the management of this important syndrome [11].

Three new classification systems were recently developed for diagnosing acute kidney injury (AKI). The first, Risk, Injury, Failure, Loss, and End-stage kidney disease (RIFLE) [12] was developed by the Acute Dialysis Quality Initiative group. RIFLE is graded in increasing levels of severity. The first level, Risk, is defined as an abrupt (within 1–7 days) and sustained (>24 h) serum creatinine (SCr) increase to ≥1.5 times the baseline SCr, a glomerular filtration rate (GFR) decrease >25% from the baseline GFR, or a urine output <0.5 mL/kg/h for >6 h.

The second classification was developed by the Acute Kidney Injury Network (AKIN) [13]. It is also graded in increasing levels of severity (AKIN 1 to AKIN 3) and uses the concept of small changes in SCr over a short period of time. The first level, AKIN 1, is defined as a SCr increase ≥0.3 mg/dL or ≥1.5-fold compared with the baseline in a time window of 48 h or a urine output <0.5 mL/kg/h for >6 h.

The third and latest classification was developed by the Kidney Disease: Improving Global Outcomes (KDIGO) Acute Kidney Injury Work Group for their freshly launched KDIGO Clinical Practice for Acute Kidney Injury [14]. This definition is a wider combination from RIFLE and AKIN criteria and defined AKI as an increase in SCr by ≥0.3 mg/dL within 48 hours or an increase in SCr to ≥1.5 times baseline, which is known or presumed to have occurred within the prior 7 days; or an urine volume <0.5 mL/kg/h for 6 hours. The combined use of small absolute and relatives increases in SCr in the KDIGO criteria may potentially make it more sensitive than RIFLE criteria. The new KDIGO criteria were not yet validated in AMI patients, and there are no data comparing AKI incidence and mortality defined by RIFLE and KDIGO after an AMI.

The aim of this study was to compare the incidence and the early (30-days) and late (1-year) mortality of AKI patients diagnosed by RIFLE and KDIGO criteria after ST-segment elevation myocardial infarction (STEMI) or non-ST-segment elevation myocardial infarction (NSTEMI).

## Methods

### Ethics Statement

This protocol was approved by the Institutional Ethics Committee (“Comitê de Ética em Pesquisa em Seres Humanos da Faculdade de Medicina de Sao José do Rio Preto”, Sao José do Rio Preto, Brazil, process 0040/2008), which agreed that informed consent was not necessary because of the purely observational nature of this study.

### Diagnostic Criteria for AKI

AKI was diagnosed and staged using the RIFLE and KDIGO SCr criteria (Table 1). GFR and urinary output criteria were not used for AKI diagnosis and staging in this study.

**Table 1:** Serum creatinine criteria for defining and staging AKI*.

Stage	Criteria	
RIFLE†		
Risk		SCr×1.5 versus baseline
Injury		SCr×2 versus baseline
Failure		SCr×3 versus baseline, or SCr≥4.0 mg/dL with an acute increase≥0.5 mg/dL
KDIGO†		
1		SCr increase 1.5–1.9 times baseline or ≥0.3 mg/dL (≥26.5 µmol/L)
2		SCr increase 2.0–2.9 times baseline
3		SCr increase 3.0 times baseline or increase in SCr to ≥4.0 mg/dL (≥353.6 µmol/L) or initiation of renal replacement therapy

AKI, acute kidney injury; RIFLE, Risk, Injury, Failure, Loss, and End-stage kidney Disease; SCr, serum creatinine. KDIGO, Kidney Disease: Improving Global Outcomes.

* Modified from references [12], [14].

† Only the SCr criteria were used to diagnose and stage AKI and therefore glomerular filtration rate and urinary output criteria were omitted.

### Patients

**Figure 1:**
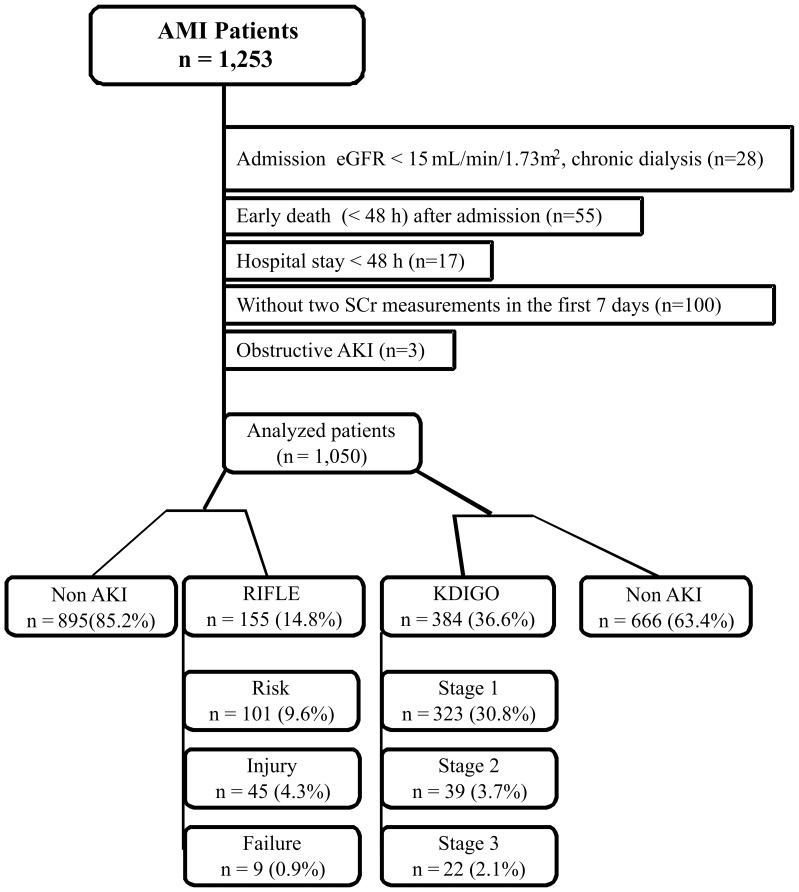
Flow chart showing the total cohort, excluded patients and stratification by AKI severity grade according to the AKI criteria used (KDIGO or RIFLE). AMI, acute myocardial infarction; SCr, serum creatinine; eGFR, estimated glomerular filtration rate; AKI, acute kidney injury; RIFLE, Risk, Injury, Failure, Loss, and End-stage kidney Disease; KDIGO, Kidney Disease: Improving Global Outcomes.

This is a single center observational prospective cohort study. In total, 1,253 consecutive patients (October 2004 to December 2009) who were diagnosed as acute STEMI or NSTEMI [15] were assessed in a prospective database on thoracic pain. Patients were included if ≥18 years-old, hospitalization >48 h and had at least two SCr measurements in the first 7 days of hospitalization. Only the first hospital admission was considered if a patient had more than one hospitalization for AMI during the study period. Reasons for exclusion are shown in figure 1.

The final studied cohort was composed of 1,050 subjects assessed by RIFLE and KDIGO criteria (Figure 1).

### Creatinine Measurements and GFR Calculation

The Jaffé colorimetric method (ADVIATM 1650, Bayer, Germany) was used for the SCr measurements. The SCr was measured on hospital admission, daily during the intensive care unit stay and thereafter as needed.

The estimated GFR (eGFR) was calculated by the Modification of Diet in Renal Disease formula (MDRD) [16].

### Systolic Left Ventricular Function (LVF) Measurements

The systolic LVF (classified as normal, mild, moderate or severe dysfunction) [17] was assessed by echocardiography, as needed, in 85.7% (900) of the patients.

### Outcomes

The primary end point was death from any cause at 30 days and at the 1-year follow-up for those patients who survived after 30 days. Events occurring after one year were censored on day 365. The follow-up after discharge was obtained by reviewing electronic hospital system records, mail, or telephone contact.

### Statistical Analysis

The baseline characteristics (presented as medians with interquartile ranges) were compared via the t-test, Mann-Whitney test or Kruskal-Wallis test followed by the Dunn post-test for continuous variables. Categorical variables (presented as numbers and percentages) were compared by χ^2^ statistics or Fisher’s exact test.

Event-free survival curves were estimated by the Kaplan-Meier method, and the curves were compared with the log-rank test in univariate analyses. Multivariate Cox proportional hazards analyses were used to assess the relationship between AKI and mortality in the first 30 days and at the 1-year follow-up, adjusting for those variables with p<0.15 in the univariate analysis for mortality in each period and considered clinically important to be controlled. The proportional hazards test and the plotted cumulative survival estimate after the ln (-ln) transformation suggested that the hazards of these variables were proportional for the period analyzed.

For Cox analyses at 30 days the model was adjusted for age (reference <65 years), gender (reference female), admission eGFR (reference ≥60 mL/min/1.73 m^2^), Killip class (reference ≤I at admission), systolic blood pressure (SBP) (reference ≥100 mmHg on admission), heart rate (HR) (reference ≤100 beats/min on admission), creatine phosphokinase (CPK-MB) and admission glycemia (continuous). The model was also adjusted for diabetes history, extracardiac vascular disease (EVD) history, clopidogrel use during hospitalization, use of diuretics, coronary angiography during hospitalization, reperfusion therapy with primary percutaneous coronary intervention (PCI) for STEMI, any type of revascularization with either PCI or coronary artery bypass graft (CABG) performed during hospitalization, reinfarction and severe systolic left ventricular dysfunction (LVD). For these variables, the reference was their absence or presence.

The multivariate Cox analysis at 1-year included all patients who survived for 30 days after AMI, with censoring at 365 days. The controlling variables were the same as those included at mortality at 30 days evaluation, except for gender, SBP<100 mmHg, CPK-MB and reinfarction which did not meet the selection criteria (p>0.15 in the univariate analysis) and for history of hypertension and prior use of angiotensin converting enzyme inhibitors (ACEIs) or angiotensin II receptor blockers (ARBs), which were also included (p<0.15 in the univariate analysis).

Differences were considered statistically significant by a two-tailed p<0.05 or based on a 95% confidence interval (CI). Analyses were performed with SPSS statistical software (version 15.0, Chicago, IL, USA).

## Results

### Study Population Characteristics

The clinical characteristics of the general population are shown in Table 2. The median for the hospitalization time was 7 (interquartile range: 4–12) days. Reinfarction occurred in 5.1% and dialysis was performed in 2.1% of the entire analyzed cohort. The in-hospital mortality rate was 13%.

**Table 2:** Baseline clinical characteristics and medical therapy during hospitalization.

Characteristics	(n = 1050 )
Age (y)	65 (55–74)*
Male	674 (64.2%)
White	969 (92.3%)
Hypertension history	730 (69.5%)
Current smoking	389 (37%)
Diabetes history	261 (24.9%)
Hyperlipidemia history	236 (22.5%)
EVD history	80 (7.6%)
Previous PCI†	93 (8.9%)
Previous CABG†	88 (8.4%)
Prior CAD (stenosis >50%)†	151 (14.4%)
Previous infarction†	153 (14.6%)
ACEs/ARBs prior use ‡	443 (42.6%)
STEMI	518 (49.3%)
Killip classes II–IV§	87 (19.3%)
Anterior wall infarction§	279 (53.9%)
SBP<100 mmHg†	67 (6.4%)
Weight (kg)	70 (62–80)*
HR >100 (beats/min)	165 (15.7%)
CK-MB (IU/L)	85 (39–187)*
Admission SCr (mg/dL)	1.2 (1.0–1.5)*
Admission eGFR (mL/min/1.73 m^2^)	61 (47–78.6)*
Admission glycemia (mg/dL)	123 (100–176)*
Medical therapy and invasive procedures	
Aspirin	1043 (99.3%)
Clopidogrel	893 (85%)
β-Blockers	985 (93.8%)
ACEIs or ARBs	1019 (97%)
Statins	973 (92.7%)
Coronary angiogram	858 (81.7%)
Any PCI||	531 (50.6%)
CABG	66 (6.3%)
Reperfusion therapy§	
Primary PCI	270 (52.1%)
Thrombolytics	150 (29%)

Continuous variables are presented as the medians (with interquartile ranges), and categorical variables are presented as numbers and percentages. EVD, extracardiac vascular disease; PCI, percutaneous coronary intervention; CABG, coronary artery bypass graft; CAD, coronary artery disease; ACEIs, angiotensin-converting enzyme inhibitors; ARBs, angiotensin II receptor blockers; STEMI, ST-segment elevation myocardial infarction; SBP, systolic blood pressure; HR, heart rate; SCr, serum creatinine; eGFR, estimated glomerular filtration rate.

* interquartile range,

† n = 1049;

‡ n = 1040;

§ n = 518 (STEMI patients);

|| primary or non-primary.

The systolic LVF was normal in 30.1%, mildly dysfunctional in 27.9%, moderately dysfunctional in 22% and severely dysfunctional in 20% of the patients who had this measurement done.

### Baseline Characteristics and Therapy during the Hospital Stay Comparing AKI to Non-AKI Patients According to the Two AKI Criteria used (Table 3)

Overall, independently of the AKI criteria used, the patients who developed AKI were older and more frequently had a history of hypertension and diabetes than non-AKI patients. A higher proportion of AKI patients were Killip class>I and had a HR >100 beat/min at admission than non-AKI subjects. AKI patients received less medical and reperfusion therapy and underwent less invasive procedures than non-AKI patients, but they received more diuretics, and a higher proportion had severe LVD. Patients who developed AKI according to KDIGO had a higher proportion of previous CABG, EVD, and prior use of ACEIs or ARBs than non-AKI patients.

**Table 3:** Baseline clinical characteristics and medical therapy during hospitalization of AKI versus non-AKI patients according to the two AKI criteria used.

Characteristics	KDIGO N = 1050			RIFLE N = 1050		
	AKI (n = 384)%	no AKI(n = 666)%	p-value	AKI (n = 155)%	no AKI (n = 895)%	p-value
Age >65 (y)	62.2	45.2	<0.001	65.2	40.1	<0.001
Male	60.9	66.1	0.095	56.1	65.6	0.023
HTN history	76.6	65.5	<0.001	78.1%	68	0.012
Current smoking	33.1	39.1	0.043	34.2	37.5	0.425
Diabetes history	33.3	20	<0.001	37.4	22.7	<0.001
HCL history	25.5	20.7	0.073	23.9	22.2	0.652
EVD history	11.5	5.4	<0.001	10.3	7.2	0.169
Prior CAD*	15.9	13.5	0.296	14.8	14.3	0.865
Previous infarction*	17.2	13.1	0.070	14.8	14.5	0.923
Previous CABG*	10.7	7.1	0.042	8.4	8.4	0.999
ACEIs/ARBs prior use †	47.6	39.7	0.013	48.1	41.6	0.138
STEMI‡	52.9	47.3	0.082	53.5	48.6	0.256
Killip classes II–IV‡	33	10.5	<0.001	36.1	16.1	<0.001
Anterior wall‡	31.1	24	0.013	32.9	25.5	0.054
SBP<100 (mm Hg)	8.3	5.3	0.050	13.5	5.1	<0.001
HR >100 (beats/min)	20.6	12.9	0.001	25.2	14.1	<0.001
Admission SCr (mg/dL)	1.2 (1.0–1.7)	1.2 (1.0–1.4)	<0.001	1.2 (0.9–1.6)	1.2 (1.0–1.5)	0.940
eGFR <60 mL/min/1.73 m^2^	53.1	45.9	0.025	52.3	47.9	0.320
Aspirin	98.7	99.7	0.107	96.8	99.8	<0.001
Clopidogrel	81.5	87.1	0.015	75.5	86.7	<0.001
β-Blockers	88.8	96.7	<0.001	83.2	95.6	<0.001
ACEIs or ARBs	94	98.5	<0.001	91.6	98	<0.001
Diuretics	77.9	44.1	<0.001	78.7	52.6	<0.001
Coronary angiography	72.9	86.8	<0.001	66.5	84.4	<0.001
Any PCI§	45.1	53.8	0.007	45.8	51.4	0.199
CABG	5.7	6.6	0.573	3.9	6.7	0.180
Primary PCI‡	42.9	58.1	0.001	46.3	53.7	0.221
Any reperfusion‡ ^||^	75.9	83.8	0.025	70.7	83.3	0.007
Severe LVD#	24.4	17.7	0.017	27.2	19	0.04

Continuous variables are presented as medians (with interquartile ranges), and categorical variables are presented as percentages. AKI, acute kidney injury; RIFLE, Risk, Injury, Failure, Loss, and End-stage kidney Disease; KDIGO, Kidney Disease: Improving Global Outcomes; HTN, hypertension; HCL, hypercholesterolemia; EVD, extracardiac vascular disease; CAD, coronary artery disease; CABG, coronary artery bypass graft; ACEIs, angiotensin-converting enzyme inhibitors; ARBs, angiotensin II receptor blockers; STEMI, ST-segment elevation myocardial infarction; SBP, systolic blood pressure; HR, heart rate; SCr, serum creatinine; eGFR, estimated glomerular filtration rate; PCI, percutaneous coronary intervention; LVD, left ventricular dysfunction.

* n = 1049;

† n = 1040;

‡ n = 518 (only for STEMI patients);

§ Primary or non-primary;

|| with PCI or thrombolytics;

# n = 9.

### Comparative Incidence, Hospitalization Length and Mortality According to Each Criteria for AKI

Incidence and stratification by AKI severity are presented in Figure 1. The higher AKI incidence found by KDIGO (36.6%) compared with RIFLE (14.8%) was the result of the larger number of patients in KDIGO stage 1 compared with the RIFLE stage Risk (30.8% vs. 9.6%, respectively).

AKI patients diagnosed by RIFLE had a greater median length of hospital stay (8.4 [interquartile range: 3.6–16.5] days versus 7.5 [interquartile range: 4.4–12.6] days, p<0.001) than patients without AKI. In the same way, patients diagnosed by KDIGO had greater median length of hospitalization (9.4 [interquartile range: 4.7–16.2] days versus 6.7 [interquartile range: 4.3–11.5] days, p<0.001) than subjects without AKI.

AKI patients diagnosed by any AKI criteria had a significantly higher 30-day and 30-days to 1-year mortality rates assessed by univariate analysis than subjects without AKI (Table 4).

**Table 4:** Univariate analyses for mortality at 30 days and 1-year and comparison between patients who developed and did not develop AKI according to the different AKI definitions.

Definition, studied n	Mortality		p-value
	AKI	No AKI	
RIFLE			
30 days (n = 1050)	38.1% (59/155)	8.0% (72/895)	<0.001
1 year* (n = 919)	24% (23/96)	10.8% (89/823)	<0.001
KDIGO			
30 days (n = 1050)	26% (100/384)	4.7% (31/666)	<0.001
1 year* (n = 919)	22.5% (68/336)	7.6% (48/635)	<0.001
AKI by KDIGO but not RIFLE			
30 days **(**n = 895)	17.9% (41/229)	4.7% (31/666)	<0.001
1 year* (n = 823)	21.9% (41/187)	7.5% (48/636)	<0.001

AKI, acute kidney injury; RIFLE, Risk, Injury, Failure, Loss, and End-stage kidney Disease; KDIGO, Kidney Disease: Improving Global Outcomes.

* 1-year mortality was estimated for those patients who survived after 30 days.

### Patients Diagnosed as AKI by KDIGO but not RIFLE Criteria

Two hundred twenty-nine patients (21.8% of the entire cohort) were diagnosed as AKI by KDIGO but not by RIFLE. These patients had a greater median length of hospital stay (9.5 [interquartile range: 5.4–16.0] days versus 6.7 [interquartile range: 4.3–11.5] days, p<0.001) than patients without AKI by both criteria.

These patients had also a higher 30-day and 30-day to 1-year mortality rates compared with patients who were non-AKI by both criteria (Table 4).

### Cox Analyses at 30 days and at 30-day to 1-year

**Figure 2:**
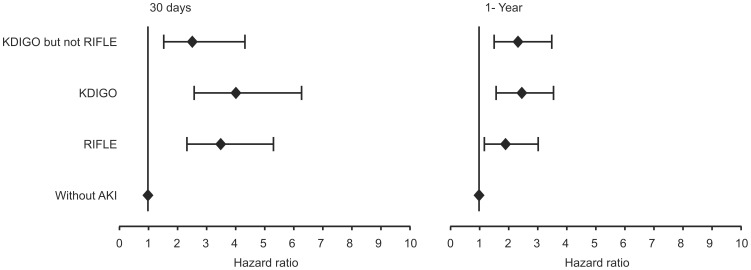
Hazard ratio (Cox multivariate analysis) for death at 30 days and at 30-day to 1-year follow-up according to the different AKI criteria. AKI, acute kidney injury; RIFLE, Risk, Injury, Failure, Loss, and End-stage kidney Disease; KDIGO, Kidney Disease: Improving Global Outcomes.

**Figure 3:**
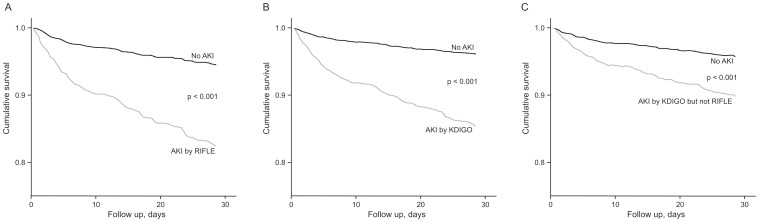
Cox survival curve at 30 days according to the different AKI criteria. A. RIFLE; B. KDIGO; C. KDIGO but not RIFLE. AKI, acute kidney injury; RIFLE, Risk, Injury, Failure, Loss, and End-stage kidney Disease; KDIGO, Kidney Disease: Improving Global Outcomes.

**Figure 4:**
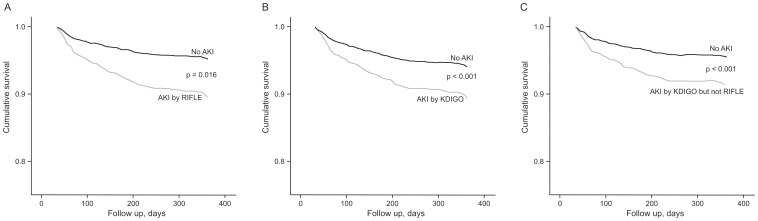
Cox survival curve at 30-days to 1-year according to the different AKI criteria. A. RIFLE; B. KDIGO; C. KDIGO but not RIFLE. AKI, acute kidney injury; RIFLE, Risk, Injury, Failure, Loss, and End-stage kidney Disease; KDIGO, Kidney Disease: Improving Global Outcomes.

The development of AKI by RIFLE or KDIGO during hospitalization assessed by Cox analyses remained independently associated with mortality at 30 days and at 30-day to 1-year follow-up. Patients who were diagnosed as AKI by KDIGO but not by RIFLE criteria also had a higher AHR ratio for early and late mortality compared with patients without AKI by any criteria (Tables 5 and 6; Figures 2, 3 and 4).

**Table 5:** Cox proportional hazard models for the association between 30-day mortality and AKI according to the different AKI criteria used.

Criteria for AKI	AHR		95% CI		p-value		
Patients without AKI*		1.0					
RIFLE	3.51		2.35–5.25		<0.001		
KDIGO	3.99		2.59–6.15		<0.001		
KDIGO but not RIFLE	2.55		1.52–4.28		<0.001		

AKI, acute kidney injury; AHR, adjusted hazard ratio; CI, confidence interval; RIFLE, Risk, Injury, Failure, Loss, and End-stage kidney Disease; KDIGO, Kidney Disease: Improving Global Outcomes.

The model was adjusted for age, gender, admission estimated glomerular filtration rate, history of diabetes, history of extracardiac vascular disease, Killip class, admission systolic blood pressure, admission heart rate, admission creatine phosphokinase and glycemia, clopidogrel use during hospitalization, therapy with diuretics, coronary angiography during hospitalization, reperfusion therapy with primary percutaneous coronary intervention (PCI) for ST-segment elevation myocardial infarction, any kind of revascularization with either PCI or coronary artery bypass graft performed during hospitalization, reinfarction or severe systolic left ventricular dysfunction.

* The AHR was estimated for each set of criteria with consideration of patients without AKI for each.

**Table 6:** Cox proportional hazard models for the association between 30-day to 1-year mortality and AKI according to the different AKI criteria used.

Criteria for AKI	AHR	95% CI	p-value
Patients without AKI*	1.0		
RIFLE	1.84	1.12–3.01	0.016
KDIGO	2.43	1.62–3.62	<0.001
KDIGO but not RIFLE	2.28	1.46–3.54	<0.001

AKI, acute kidney injury; AHR, adjusted hazard ratio; CI, confidence interval; RIFLE, Risk, Injury, Failure, Loss, and End-stage kidney Disease; KDIGO, Kidney Disease: Improving Global Outcomes.

The model was adjusted for age, admission estimated glomerular filtration rate, history of hypertension and diabetes, history of extracardiac vascular disease, prior use of angiotensin-converting enzyme inhibitors or angiotensin II receptor blockers, admission Killip class, admission heart rate, admission glycemia, clopidogrel use during hospitalization, therapy with diuretics, coronary angiography during hospitalization, reperfusion therapy with primary percutaneous coronary intervention (PCI) for ST-segment elevation myocardial infarction, any type of revascularization with either PCI or coronary artery bypass graft performed during hospitalization or severe systolic left ventricular dysfunction.

* The AHR was estimated for each set of criteria, considering patients without AKI for each.

## Discussion

In the best of our knowledge there are no previous data assessing and comparing AMI-associated AKI diagnosed by RIFLE and KDIGO criteria [18]. KDIGO had a higher detection rate for AKI than RIFLE criteria and was the most inclusive criteria. Importantly, patients who were diagnosed as AKI by KDIGO but were missed by RIFLE criteria had a significantly longer hospitalization and a significantly higher AHR for death, which has an obvious clinical implication.

### Differences in the AKI Detection Rate by RIFLE Compared with KDIGO

KDIGO diagnosed more AKI than RIFLE during the first seven days of hospitalization. When compared with RIFLE the KDIGO definition diagnosed AKI in an additional 229/1,050 patients, who represented 21.8% of the entire analyzed cohort. If only RIFLE criteria were used, a significant number of patients would be misclassified as non AKI by RIFLE.

The previously reported incidence of AMI-associated AKI is extremely heterogeneous, ranging from 5 to 55% and varies with the criteria used for diagnosing AKI and the studied clinical setting [1]–[10], [19], [20]. These large disagreements in the incidence of AKI among previous studies reflect the lack of standardization for diagnosing AKI and support the need for a defined and validated set of AKI criteria instead of arbitrary definitions.

In this study we have used only the SCr criteria for diagnosing and staging AKI by RIFLE definition, although the original criteria also used GFR decreases for AKI diagnosis and staging [12]. In fact, the use of GFR criteria calculated as 25% decrease for risk and as 75% decrease for failure stages has been questioned since in steady-state the 1.5-fold increase in SCr would correspond to a one-third decrease (instead of 25%) in GFR and the 3-fold increase in SCr would correspond to a two-thirds decrease (instead of 75%) in GFR [21]. Moreover, GFR is seldom measured in the context of AKI, but estimated by formulas using SCr measurements. Nevertheless, estimation of GFR by formulas is only valid when SCr is in equilibrium, which is not the case for AKI.

We also decided not to use the urinary output criteria, since they are less validated, may be influenced by several drugs used by this studied population and are very difficult to capture and measure reliably in the emergency room setting.

### Why is the AKI Detection Rate Higher with KDIGO than RIFLE?

The most relevant difference between KDIGO and RIFLE is related to the conditions necessary to classify a patient as KDIGO stage 1 or RIFLE stage Risk. Whereas a SCr increase of ≥50% from baseline is necessary in RIFLE, by the KDIGO definition, only an absolute SCr increase of 0.3 mg/dL (≥26.5 µmol/L) within 48 hours is sufficient for an AKI diagnosis. The 0.3 mg/dL variation was included in the KDIGO definition because such small changes in SCr have been independently associated with death in AKI patients [22], [23]. Small changes in SCr have also been associated with early and long-term mortality in cohorts of AMI patients [3], [4].

A potential problem with the use of a moving 48 h window for AKI diagnosis, as recommended by KDIGO and AKIN definitions, is when SCr decreases in comparison with the pre-event value due to fluid accumulation with a subsequent increase of at least 0.3 mg/dL. In a study on AKI after cardiopulmonary bypass the authors found significantly more patients diagnosed as AKI by AKIN (26.3%) than by RIFLE (18.9%) criteria (p<0.0001). Patients classified as AKI in one but not in the other definition set were predominantly staged in the lowest AKI severity class (9.6% of patients in AKIN stage 1 and 2.3% of patients in RIFLE class R). The authors determined that the differences between patients diagnosed as AKI by RIFLE or AKIN were mainly found in the subgroup that had an initial decrease of SCr from preoperative baseline to post-operatory day 1, probably due to fluid accumulation during surgery. In this subgroup, post-operative SCr values that were lower than preoperative levels served as comparison in the 48-hour moving diagnostic window of AKIN [24]. That is not the case for the current study since patients after AMI are not massively hydrated as during cardiopulmonary bypass, and consequently the AMI patients assessed are certainly less affected by a dilution of the baseline SCr. Although our data are favorable for the use of the KDIGO criteria, the reference SCr possibly needs to be differently defined depending on the particular studied population, and in some circumstances the pre-event baseline SCr could be more reliable for AKI diagnosis than the moving 48 hour SCr window.

### Mortality

After controlling for the prognostic variables that might affect mortality after STEMI and NSTEMI, AKI diagnosed by both criteria remained independently associated with mortality at 30-days and 1-year follow-up.

The most important novel finding of the current study is that patients with an AKI diagnosis by KDIGO, but not by RIFLE, had a higher AHR for mortality when compared with non-AKI patients. These results indicate that a large number of patients with AMI-induced AKI and elevated risk for mortality would be missed by the RIFLE criteria.

### Study Limitations

This was an observational single-center prospective cohort study.

A possible study limitation is related to the time of the assessment of the reference SCr level. This limitation occurred due to difficulties to obtain reference SCr data prior to the event precipitating admission, as most patients have not undergone a previous renal function evaluation prior to their hospital entry for an acute illness as AMI. Various strategies for dealing with pre-event absent reference SCr have been highlighted, such as using lowest admission or discharge SCr against which to diagnose AKI [25]. In the current study we have chosen to use the admission SCr as the reference value used to diagnose AKI. As a result, it is possible that some patients who were hospitalized already had AKI, but because the SCr did not increase further, they were misclassified as non-AKI. In the same way, patients who present with renal dysfunction at admission and theirs SCr did not decrease after admission actually might have suffered an AKI just before the hospitalization. In fact, a recent study on cardiac arrest patients requiring resuscitation suggested that patients whose SCr did not decrease after admission in that scenario, actually did have AKI [26].

## Conclusions

KDIGO had a higher detection rate for AKI than RIFLE. Patients misclassified as non-AKI by RIFLE had a higher AHR for death when compared with non-AKI subjects according to any definition. As a more inclusive criterion, KDIGO is probably more suitable for AKI diagnosis and risk stratification in patients with AMI.
